# Nocturnal respiratory support with nasal high flow in hypercapnic COPD: a randomised, crossover trial

**DOI:** 10.1183/23120541.01063-2024

**Published:** 2025-06-30

**Authors:** Georg Nilius, Maik Schroeder, Ulrike Domanski, Mohamed Khalaf, Stanislav Tatkov

**Affiliations:** 1 Klinikum Dortmund gGmbH, Dortmund, Germany; 2 University Witten/Herdecke, Witten, Germany; 3 KEM Evang. Kliniken Essen-Mitte gGmbH, Essen, Germany; 4 Vamed Klinik Hagen-Ambrock, Hagen, Germany; 5 Fisher & Paykel Healthcare Limited, Auckland, New Zealand

## Abstract

**Background:**

Nasal high flow (NHF) is an established treatment option in acute respiratory failure and has been shown to increase the elimination of carbon dioxide (CO_2_) in hypercapnic COPD patients. The aim of the study was to investigate the impact on gas exchange, respiratory pattern and sleep quality in severe COPD patients with mild hypercapnic respiratory failure.

**Methods:**

Hypercapnic COPD patients (n=42) underwent a wakefulness ventilation study with calibrated inductance plethysmography followed by two polysomnography (PSG) studies with NHF at 20 L·min^−1^ and 35 L·min^−1^. In a crossover design immediately after hospital discharge, patients were randomised to a 4-week period of nocturnal NHF *versus* long-term oxygen therapy (LTOT). The primary outcome was transcutaneous carbon dioxide (*P*_tcCO_2__) measured during PSG after each period.

**Results:**

NHF reduced mean overnight *P*_tcCO_2__ by 3.4±6.5 mmHg (p=0.010), attenuating the increase of CO_2_ during sleep, with no effect observed during wakefulness when minute ventilation (*V*′_E_) and respiratory rate (RR) were decreased. The mean sleep fragmentation index (26.6±11.2) and sleep efficiency (72.8±16.4%) during NHF were not significantly different from those during LTOT (n=24). An increase in NHF did not change *V*′_E_ during wakefulness and did not produce significant effects on ventilation, gas exchange or sleep parameters during the night.

**Conclusion:**

Nocturnal NHF attenuated an increase of *P*_tcCO_2__ during sleep in hypercapnic COPD patients without a relevant effect on sleep quality compared with LTOT. During wakefulness, *P*_tcCO_2__ was unaffected, but *V*′_E_ and RR were reduced, indicating a different physiological response during wakefulness compared with sleep.

## Shareable abstract

**In patients with mild hypercapnic COPD, nocturnal NHF did not affect sleep quality but did mitigate sleep-related increase in *P*_tcCO_2__. During wakefulness, NHF did not affect *P*_tcCO_2__ or *P*_aCO_2__, but it reduced ventilation, indicating a lower work of breathing.** https://bit.ly/4gK5IRJ

## Introduction

COPD demonstrates increased morbidity and mortality worldwide, while chronic hypercapnic COPD patients have a particularly poor prognosis [1–3]. Chronic hypercapnia is associated with various effects such as epithelial dysfunction, changes in lung immunity and muscle atrophy [4]. Long-term oxygen therapy (LTOT) has shown improvement in health outcomes and mortality in chronic hypoxaemic COPD patients [5]. Noninvasive ventilation (NIV) offers a therapeutic option for hypercapnic COPD patients but is still the subject of controversial debates [6–10]. Nasal high flow (NHF) of heated and humidified air up to 70 L·min^−1^ with or without supplemental oxygen through a nasal cannula interface is extensively used in a diverse patient population, but there is no universal guidance on the flow-rate selection, and higher NHF settings have been suggested for patients with increased breathing frequency [11, 12]. In stable patients, lower NHF rates are usually chosen to improve compliance, as NHF above 35 L·min^−1^ may disrupt sleep [13]. Patients in a state of acute COPD exacerbation might accommodate higher flow rates, but it is reasonable to assume that adherence to long-term high-flow therapy may be better when the flow rate is less disturbing. NHF therapy in physiological studies has demonstrated a reduction of increased respiratory rate (RR). A decrease in both minute ventilation (*V*′_E_) and the work of breathing (WOB) has been reported, as well as improved oxygenation and reduced hypercarbia [14, 15]. It was shown that NHF improved exercise capacity and reduced hyperinflation in COPD patients [16–18]. As LTOT can only correct hypoxaemia and can be associated with increased carbon dioxide (CO_2_) levels, NHF could provide respiratory support in patients with increased respiratory mechanical loads [17, 19]. A flow-dependent reduction of the CO_2_ level by NHF was found in chronic hypercapnic COPD patients [20]. A reduction of arterial CO_2_ by NHF in COPD patients after 6 weeks of home treatment has been demonstrated, while the mechanism of these beneficial effects remains unknown [12, 21, 22].

This study investigated the physiological effects on gas exchange in wakefulness and sleep in hospital and at home during nocturnal respiratory support with NHF in hypercapnic COPD patients in a crossover design. The investigators tested a hypothesis that nocturnal NHF in COPD patients with chronic hypercapnic respiratory failure, initiated immediately after severe acute exacerbation, improves ventilation and gas exchange, leading to a reduction of accumulated CO_2_ without a negative effect on sleep.

## Methods

### Study design

This single-centre, open-label, prospective, randomised, crossover trial was registered under ClinicalTrials.gov NCT02083120 and approved by the ethics research committee of University Witten/Herdecke, Germany under Nr. 13/2014. The trial investigated gas exchange, ventilation during wakefulness, sleep quality, life quality and exercise capacity during hospitalisation and after 4 weeks of either NHF or LTOT at home. The main inclusion criteria were hospitalised COPD patients due to acute exacerbation, chronic hypercapnia with arterial carbon dioxide tension (*P*_aCO_2__) >50 mmHg and pH >7.35, and age between 40 and 80 years. Patients were excluded from the study if *P*_aCO_2__ values dropped below 50 mmHg at any time during inpatient stay. Further exclusion criteria included: known or apparent obstructive sleep apnoea (OSA); usage of NIV; decompensated heart, liver or kidney failure; pregnancy or nursing period; participation in another clinical trial; drug abuse; and incapability of giving consent. All prescribed medications were continued. Baseline data included lung function, blood gas analysis before and after the 6-min walk test (6MWT), COPD Assessment Test (CAT) and St. George's Respiratory Questionnaire (SGRQ).

**FIGURE 1:**
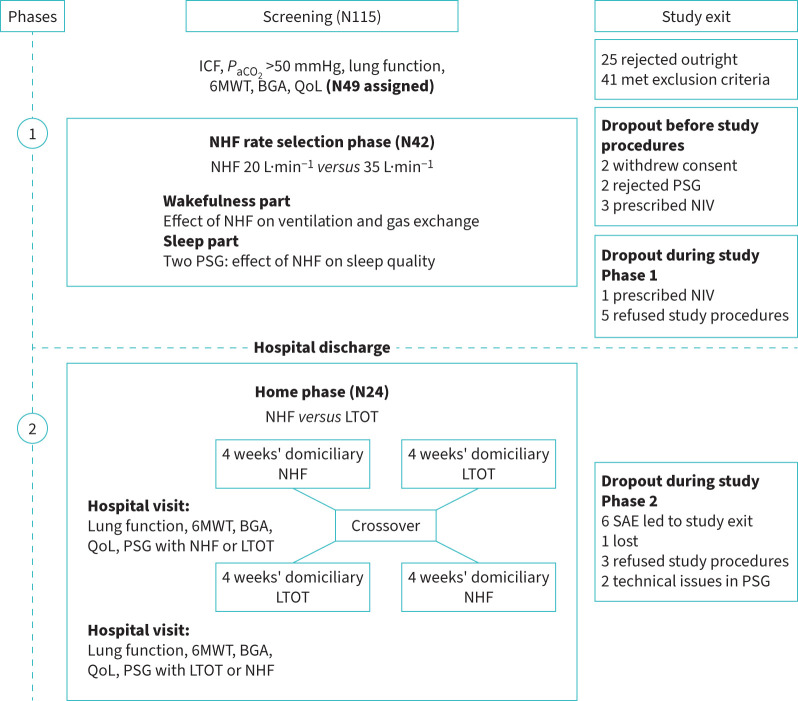
Study flow chart and study exit. NHF: nasal high flow; LTOT: long-term oxygen therapy; NIV: noninvasive ventilation; 6MWT: 6-minute walk test; QoL: quality of life; PSG: polysomnography; *P*_aCO_2__: arterial carbon dioxide tension; BGA: blood gas analyses; ICF: informed consent form; SAE: serious adverse events.

The study comprised two phases. The first phase, focused on the NHF rate selection and physiological effects of NHF during wakefulness and sleep, was completed before discharge. The second phase involved home-based nocturnal NHF therapy, and patients were assessed during hospital visits in between (see figure 1). The primary outcome was the mean overnight transcutaneous carbon dioxide (*P*_tcCO_2__) following the domiciliary night-time NHF *versus* LTOT over a 4-week period.

### NHF rate selection

During daytime, physiological parameters were measured in a semi-recumbent position during random application of NHF (Optiflow™, myAIRVO™ 2, Fisher & Paykel Healthcare, New Zealand) at rates of 20 and 35 L·min^−1^ (30 min each period) with supplemental oxygen to maintain peripheral arterial oxygen saturation (*S*_pO_2__) between 88% and 92%. Ventilation was monitored by respiratory inductance plethysmography (Respitrace-QDC, CareFusion, USA) calibrated with a pneumotachometer (Hans Rudolph, USA). *P*_tcCO_2__ and *S*_pO_2__ were monitored with TOSCA (Radiometer, Denmark). The data were recorded and subsequently analysed using PowerLab and LabChart software (ADInstruments, New Zealand) [23].

NHF was then randomly applied with either 20 L·min^−1^ or 35 L·min^−1^ over two consecutive nights. Supplemental oxygen was adjusted during instrumentation before the sleep and was not changed during the night. Sleep parameters were recorded with unattended polysomnography (PSG) (Alice 4, Philips/Respironics, Murrysville, USA) including *P*_tcCO_2__ and *S*_pO_2__. An elimination efficiency index of CO_2_ was calculated from the volumetric capnography [24].

### Home study

When patients were discharged, they were randomised into two groups with 4-week periods of LTOT or NHF in a crossover design. The patients were advised to use the NHF overnight. After each treatment period, patients visited the hospital for an overnight PSG and to test the lung function, 6MWT and health-related quality of life (QoL) by CAT and SGRQ. PSG was performed with either LTOT or NHF depending on the completed domiciliary period.

### Statistical analyses

The sample size estimation, based on a previous study, indicated a calculated case number of 37 patients assuming a difference in mean *P*_tcCO_2__ overnight of 2 mmHg with α 0.05 and a power of 90% [22]. The data were analyzed using STATISTICA (Statsoft Europe GmbH, Hamburg, Germany). Descriptive statistics were used for all obtained data, evaluating mean values and standard deviation. Comparative statistics using the Wilcoxon test were employed to evaluate the primary objectives in this randomised crossover study. For the main and secondary target parameters, an analysis of variance (ANOVA) with repeated measures (LTOT, NHF) and one of the potential predictors as an independent factor was performed in each case.

## Results

### Patient selection and drop outs

In total, 115 hospitalised hypercapnic COPD patients recovering from exacerbation were initially screened, of which 66 were ineligible (25 declined, 12 were diagnosed with OSA, eight used NIV, 10 patient's CO_2_ values dropped below 50 mmHg and 11 suffered from other comorbidities). 49 patients were ultimately enrolled, and seven dropped out before study measurements began (two withdrew consent, two rejected PSG, three used NIV). 42 patients completed the first phase of PSG recordings, and their anthropometric and clinical baseline data before discharge are presented in table 1. Four daytime recordings failed due to technical reasons and two patients refused the daytime measurements (but agreed to participate in the domiciliary phase), leading to 36 cases in the wakefulness ventilation part.

**TABLE 1:** Anthropometric and clinical baseline data of patients before discharge

**Female**	24
**Male**	18
**Age years**	66.8±7.2
**Height cm**	164.6±7.9
**Weight kg**	64.3±18.3
**BMI kg·m^−2^**	23.7±6.3
***P*_aCO_2__ initial mmHg**	56.7±6.1
**Lung function**	
** **FEV_1_ absolute	0.7±0.2
** **FEV_1_ % predicted	28.5±7.5
** **FEV/FVC %	51.4±12.2
** **FVC % predicted	54.6±14.7
** **RV % predicted	251.7±73.3
** **TLC % predicted	140.4±42.8
** **RV/TLC %	182.1±20.5
** ***D*_LCOsb_ %	19.3±16.3
**Before discharge**	
** **6MWT m	210.5±134.8
** **BORG before 6MWT	2.8±1.7
** **BORG after 6MWT	6.2±2.2
** **pH	7.4±0.0
** ***P*_aO_2__ mmHg	64.8±10.9
** ***P*_aCO_2__ mmHg at rest	54.1±6.6
** ***P*_aCO_2__ mmHg 6MWT	55.8±7.3
** **Stand. bicarbonate mEq·L^−1^	30.6±3.2
** **Base excess mmol·L^−1^	6.9±3.3
** ***S*_pO_2__ %	90.9±3.6
** **CAT total score	26.8±6.7
** **SGRQ total score	67.0±14.2

Data are presented as n or mean±sd. BMI: body mass index; *P*_aCO_2__: arterial carbon dioxide tension: FEV_1_: forced expiratory volume in 1 s: FVC: forced vital capacity: RV: residual volume: TLC: total lung capacity: *D*_LCOsb_: single breath diffusing capacity of carbon monoxide: 6MWT: 6-minute walk test: *P*_aO_2__: arterial oxygen tension: *S*_pO_2__: peripheral arterial oxygen saturation: CAT: COPD Assessment Test: SGRQ: St. George's Respiratory Questionnaire.

During the further course of the study, 18 patients dropped out (two through technically insufficient PSG recordings, eight rejected other elemental study procedures, one used NIV, one was lost to follow-up and six suffered serious adverse events (SAEs) leading to study exit). 12 dropouts occurred after being randomised to the home phase, of which 6 occurred due to acute COPD exacerbation (four LTOT, two NHF). Severe exacerbations were also considered SAEs. Two more exacerbations occurred during LTOT but these did not lead to exits from the study. 24 patients completed the home phase. Details on dropouts and SAEs, clinical data of phase 2 patients and a comparison with the protocol group can be found in the supplemental material (supplementary tables S1 and S2). On average, the patients spent 9.8+5.6 days in hospital.

### Inpatient measurements

Physiological measurements during wakefulness revealed a significant reduction in *V*′_E_ and RR by NHF, while *P*_tcCO_2__ levels remained unchanged (table 2). An increase in NHF from 20 L·min^−1^ to 35 L·min^−1^ was associated with a significant increment in mean tidal volume (*V*_T_) and a reduction in RR, but no change in *V*′_E_.

**TABLE 2:** Effect of nasal high flow (NHF) rates 20 L·min^−1^ and 35 L·min^−1^ on ventilation and gas exchange during wakefulness in Phase 1

	Baseline, mean±sd	NHF 20 L·min^−1^, mean±sd	NHF 35 L·min^−1^, mean±sd	Baseline *versus* 20 L·min^−1^ p-value	Baseline *versus* 35 L·min^−1^ p-value	20 L·min^−1^ *versus* 35 L·min^−1^ p-value
*t*E **s**	2.09±0.7	2.49±0.9	2.72±1.1	**<0.001**	**<0.001**	**0.007**
*t*I **s**	1.29±0.5	1.44±0.5	1.69±0.7	0.010	**<0.001**	**0.001**
***V*′_E_ L·min^−1^**	6.8±2.4	6.1±2.1	6.0±2.2	**<0.001**	**0.004**	0.814
***V***_**T**_ **mL**	375.0±159.8	385.7±162.1	428.8±189.6	0.683	**0.002**	**0.001**
**RR breaths·min^−1^**	19.6±5.2	17.1±4.9	15.5±4.7	**<0.001**	**<0.001**	**0.001**
***S***_**pO_2_**_ **%**	94.4±3.1	93.9±2.5	93.2±2.8	0.159	**0.011**	**0.007**
***P*_tcCO_2__ mmHg**	51.8±7.3	52.2±6.4	52.5±6.4	0.756	0.342	0.850

Data are presented as mean±sd, unless otherwise stated. NHF reduced respiratory rate and minute ventilation while *P*_tcCO_2__ was not affected. An increase of NHF rate from 20 L·min^−1^ to 35 L·min^−1^ reduced the respiratory rate and increased tidal volume without change of minute ventilation. Bold type for p-values denotes statistical significance. *t*E: expiratory time: *t*I : inspiratory time: *V*′_E_ : minute ventilation: *V*_T_: tidal volume: RR: respiratory rate: *S*_pO_2__: peripheral arterial oxygen saturation: *P*_tcCO_2__: transcutaneous carbon dioxide.

The results of the nocturnal PSG recordings are presented in table 3, and the analysis did not reveal significant differences in sleep, RR, *P*_tcCO_2__ and *S*_pO_2__ between NHF 20 L·min^−1^ and 35 L·min^−1^.

**TABLE 3:** Polysomnography during Phase 1 performed before discharge: night with nasal high flow (NHF) 20 L·min^−1^ *versus* night with NHF 35 L·min^−1^

	NHF 20 L·min^−1^, mean±sd	NHF 35 L·min^−1^, mean±sd	p-value
**TIB min**	368.1±63.3	376.5±51.9	0.688
**SPT min**	311.8±74.3	312.4±61.5	0.488
**TST min**	240.5±78.2	231.3±70.9	0.294
**WASO min**	71.2±49.4	82.2±55.8	0.274
**Sleep efficiency %**	76.5±16.0	74.2±17.7	0.345
**Stage N1 %**	18.9±20.0	17.1±16.8	0.613
**Stage N2 %**	46.3±16.5	46.9±15.1	0.970
**Stage N3 %**	26.2±35.6	24.2±15.1	0.199
**Stage REM %**	13.8±9.5	11.7±8.5	0.382
**AHI**	5.1±8.8	4.6±5.3	0.372
**Arousal Index**	29.7±17.4	31.4±15.8	0.448
**Mean *S***_**pO_2_**_ **NREM %**	89.5±4.5	89.0±3.6	0.307
**Mean *S***_**pO_2_**_ **REM %**	87.7±5.7	85.8±7.1	0.225
**RR Total breaths·min^−1^**	20.2±4.8	20.3±4.6	0.740
**RR Wake breaths·min^−1^**	19.4±3.9	19.0±3.7	0.375
**RR REM breaths·min^−1^**	19.2±4.2	19.3±4.0	0.544
**RR NREM breaths·min^−1^**	20.5±5.3	20.8±5.1	0.127
***P*_tcCO_2__ Total mmHg**	59.5±9.1	58.7±8.6	0.240
***P*_tcCO_2__ Wake mmHg**	56.8±9.0	55.9±8.4	0.215
***P*_tcCO_2__ REM mmHg**	63.6±9.7	61.8±9.7	0.090
***P*_tcCO_2__ NREM mmHg**	60.6±9.2	59.9±9.1	0.355

Data are presented as mean±sd. Patients demonstrated reduced sleep quality but no significant difference between NHF rates. TIB: time in bed; SPT: sleep period time; TST: total sleep time; WASO: wake after sleep onset; REM: rapid eye movement sleep stage; AHI: apnea–hypopnea index; *S*_pO_2__: peripheral arterial oxygen saturation; NREM: non-rapid eye movement sleep stage; RR: respiratory rate; *P*_tcCO_2__: transcutaneous carbon dioxide.

### Home study

Following the first phase, 20 out of 24 patients tolerated an NHF rate of 35 L·min^−1^ for the home phase in the treatment arm, and four preferred 20 L·min^−1^. Analysis of logged data from the devices revealed a mean NHF therapy usage at home of 5.5 h per night. PSG data comparing NHF and LTOT as well as clinical data collected during hospital visits are presented in table 4.

**TABLE 4:** Polysomnography and clinical data after 4-week treatment periods in home phase assessed during hospital visits: nasal high flow (NHF) *versus* long-term oxygen therapy (LTOT)

	NHF, mean±sd	LTOT, mean±sd	p-value
**TIB min**	398.2±35.3	375.5±53.2	0.084
**SPT min**	332.9±59.7	311.9±70.5	0.265
**TST min**	246.6±85.7	245.2±80.2	0.841
**WASO min**	87.7±49.2	67.1±44.2	0.079
**Sleep efficiency %**	72.8±16.4	77.6±15.8	0.123
**Stage N1 %**	20.9±16.5	14.1±9.7	**0.043**
**Stage N2 %**	47.1±15.1	49.2±15.4	0.549
**Stage N3 %**	21.0±14.5	22.0±13.3	0.465
**Stage REM %**	11.0±7.9	14.7±9.9	0.145
**SFI n·h^−1^**	26.6±11.2	21.4±9.9	0.103
**AHI**	3.7±4.9	4.1±5.0	0.368
**Arousal Index**	37.1±15.7	34.2±13.8	0.568
**mean *S***_**pO_2_**_ **NREM %**	90.3±4.0	94.0±4.2	**0.002**
**mean *S***_**pO_2_**_ **REM %**	89.7±5.0	93.1±4.2	**0.033**
**RR Total breaths·min^−1^**	20.1±4.6	18.8±4.3	**0.026**
**RR Wake breaths·min^−1^**	19.2±3.9	19.1±3.5	0.749
**RR REM breaths·min^−1^**	19.0±3.6	18.1±4.7	0.131
**RR NREM breaths·min^−1^**	20.7±4.6	18.7±4.6	**0.001**
***P*_tcCO_2__ Total mmHg**	54.5±7.2	57.9±8.3	**0.010**
***P*_tcCO_2__ Wake mmHg**	52.8±6.9	55.1±8.1	0.073
***P*_tcCO_2__ REM mmHg**	58.6±8.1	63.1±8.9	**0.021**
***P*_tcCO_2__ NREM mmHg**	55.6±7.6	59.3±7.9	**0.003**
**Clinical data collected during visits**			
** **6MWT m	281.3±101.3^#^	267.0±121.5^¶^	0.366
** **FEV_1_ L	0.809±0.438	0.794±0.390	0.280
** **FEV_1_ %	33.7±15.0	34.0±14.2	0.715
** **RV/TLC %	166.9±25.623.8^+^	168.9±23.8^§^	0.198
** ***P*_aCO_2__ at rest	50.7±9.2	52.1±9.0	0.200
** **BORG at rest	2.7±1.3	2.6±1.5	0.754
** ***P*_aCO_2__ 6MWT	52.3±10.1	53.5±9.6	0.349
** **BORG 6MWT	5.3±2.0	5.4±1.8	0.657
** **CAT total	20.4±6.9	24.0±8.3	0.015
** **SGRQ total	55.5±15.1	64.9±16.5	0.0001

Data are presented as mean±sd. TIB: time in bed; SPT: sleep period time; TST: total sleep time; WASO: wake after sleep onset; REM: rapid eye movement sleep stage; SFI: sleep fragmentation index; AHI: apnea–hypopnea index; *S*_pO_2__: peripheral arterial oxygen saturation; NREM: non-rapid eye movement sleep stage; RR: respiratory rate; *P*_tcCO_2__: transcutaneous carbon dioxide; 6MWT: 6-min walk test; FEV_1_: forced expiratory volume in 1 s; TLC: total lung capacity; CAT: COPD Assessment Test; SGRQ: St. George's Respiratory Questionnaire. Significant difference *versus* baseline (table 1) – **^#^**: p=0.005; ^¶^: p=0.006; **^+^**: p=0.017; ^§^: p=0.046.

**FIGURE 2:**
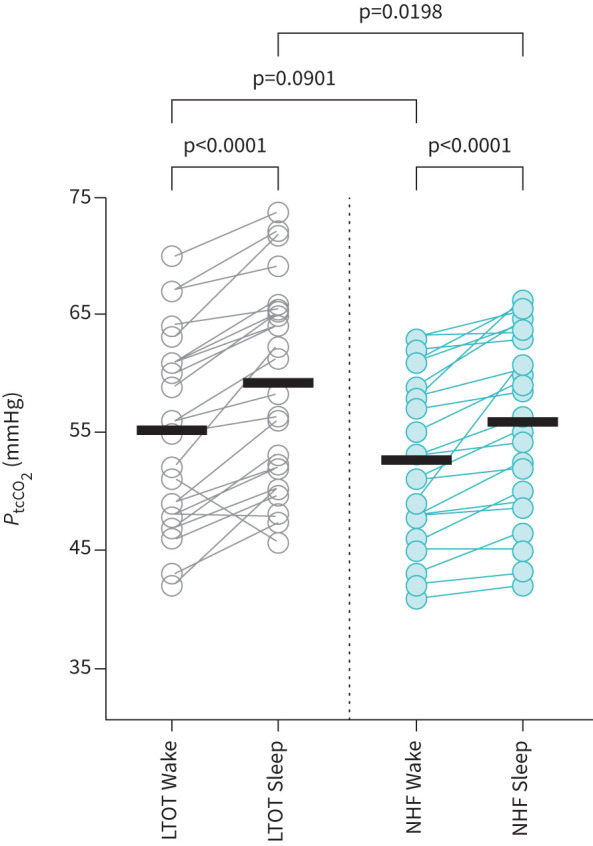
Effect of nasal high flow (NHF) and long-term oxygen therapy (LTOT) on transcutaneous carbon dioxide (*P*_tcCO_2__) during wakefulness (Wake) and sleep (non-rapid eye movement (NREM) and rapid eye movement (REM)) stages (Sleep) assessed overnight by polysomnography in the second phase. The graphs show individual values by symbols and means by horizontal lines. During sleep, *P*_tcCO_2__ was significantly increased, which was significantly attenuated by NHF.

The mean overnight *P*_tcCO_2__ values measured during PSG in the NHF group were significantly lower compared with the LTOT group (NHF: 54.5±7.2 mmHg, LTOT: 57.9±8.3 mmHg; p=0.01). *P*_tcCO_2__ was significantly higher during non-rapid eye movement sleep (NREM) and also during REM sleep stages compared with wakefulness in both groups (NREM: NHF p=0.00006; LTOT p=0.00002; and REM: NHF p=0.00013; LTOT p=0.00004). The mean *P*_tcCO_2__ was significantly lower under NHF compared with LTOT during sleep, but not during wakefulness (see figure 2).

Arterial carbon dioxide tension (*P*_aCO_2__) measured during the day did not change from the baseline measurement taken before discharge, nor after the NHF and LTOT treatment periods. To exclude time-related bias in CO_2_ reduction, a chronological analysis of CO_2_ values was performed. This analysis indicated effective randomisation, with mean *P*_tcCO_2__ at 56.1±8.1 mmHg (*P*_aCO_2__ 51.3±8.8) at the first visit and a *P*_tcCO_2__ of 56.3±7.8 mmHg (*P*_aCO_2__ 51.6±9.4) at the second control visit (see supplementary table S3).

The mean group RR in the NHF group increased significantly during the NREM stage of sleep (NHF 20.7±4.6 breaths·min^−1^, LTOT 18.7±4.6 breaths·min^−1^; p=0.001) but the RR did not change significantly in the LTOT group, although in both groups half of the patients had increased RR and half decreased. Also, the RR in both groups did not correlate with *P*_tcCO_2__ (r=0.221, p=0.132). In the LTOT group, mean *S*_pO_2__ was significantly higher than in the NHF group, and this did not correlate with *P*_tcCO_2__ (r= −0.229, p=0.118). The mean sleep fragmentation index (26.6±11.2) and sleep efficiency (72.8±16.4%) during NHF were not different from LTOT, but the mean proportion of stage 1 sleep was significantly longer (NHF: 20.9±16.5% *versus* LTOT: 14.1±9.7%, p=0.043).

Both questionnaires showed a significant improvement after the NHF treatment arm (CAT score difference NHF-LTOT: 3.5±6.6; p=0.015; SGRQ score difference NHF-LTOT: 9.4±7.6; p<0.001) (supplementary figure S1 and S2). The Spearman rank correlation for the primary outcome parameter (reduction in overnight *P*_tcCO_2__) showed a weak correlation with the degree of hyperinflation measured by the residual volume (RV)/total lung capacity (TLC) ratio (r= −0.476, p=0.029) and a positive weak correlation with both CAT and SGRQ (CAT: r=0.307, p=0.033; SGRQ: r=0.290, p=0.045). No further correlations were found. The ANOVA in search of predictors showed a significant relation between patients with a higher degree of hyperinflation (RV%TLC) and *P*_tcCO_2__ (p=0.023).

## Discussion

The study investigated the effects of nocturnal NHF on gas exchange, respiratory pattern and sleep in a group of COPD patients with mild hypercapnia following a severe exacerbation. The investigation revealed a significant reduction in mean overnight *P*_tcCO_2__ with NHF compared with LTOT following the 4-week domiciliary treatment periods. Reduction of *P*_tcCO_2__ was significant throughout all stages of sleep but not during wakefulness including the short-term daytime physiological study, wakefulness periods during PSG and *P*_aCO_2__ measured before discharge and during each hospital visit in the second phase. Sleep quality in these patients remained unaffected by NHF.

A physiological investigation performed during wakefulness by NHF over the 30-min intervention period revealed a reduction of RR in a flow-dependent manner, which was opposite to NREM sleep where the mean RR was slightly elevated. The increase in RR was not observed during the REM sleep stage. During wakefulness, NHF decreased *V*′_E_ and RR, but an increase in the NHF rate from 20 L·min^−1^ to 35 L·min^−1^ did not further reduce *V*′_E_ despite the lower RR due to a proportional increase in *V*_T_. The decreased *V*′_E_ during NHF measured in wakefulness may indicate reduced WOB, which may play a key role in improving patients with chronic respiratory failure. A higher NHF rate (35 L·min^−1^) during the night in the first phase did not affect any physiological parameters, including sleep and *P*_tcCO_2__. However, most patients tolerated a higher flow setting in the second phase.

Sleep PSG studies performed in the second phase showed a significant reduction in mean overnight *P*_tcCO_2__, with NHF being 3.4 mmHg lower compared with LTOT. The investigation revealed an increase in hypercapnia during sleep, which was partially attenuated by NHF. There was also a small but significant difference in mean *S*_pO_2__, with a lower value in the NHF group. This can be explained by a more consistent fraction of oxygen during NHF, due to reduced entrainment of room air, resulting in tighter control of *S*_pO_2__ overnight. While hyperoxia is known to increase CO_2_ in patients with hypercapnia [25], it is unlikely that this affected *P*_tcCO_2__, as no positive correlation between *S*_pO_2__ and *P*_tcCO_2__ was observed in the current study. Supplementation with pure oxygen at low flow results in dilution by entrained room air, leading to variable inspiratory oxygen fraction (*F*_IO_2__) depending on the breathing pattern. Consequently, the beneficial role of premixed inspired oxygen during NHF, which reduces room air entrainment and delivers a stable *F*_IO_2__, preventing fluctuations of *S*_pO_2__ hyperoxia regardless of variations in inspiratory flow, cannot be entirely excluded. The reduction of *P*_tcCO_2__ during sleep was significantly more pronounced in patients with higher lung hyperinflation, but no statistical correlations were found between other physiological measurements.

COPD is a very complex disease. Ultimately, hypercapnia can be attributed to an imbalance between CO_2_ production and alveolar ventilation, with heterogeneous underlying pathophysiological mechanisms [4]. Subgroup analyses were carried out to examine *P*_tcCO_2__ reduction in relation to lung function, diffusion capacity, body mass index and CAT scores, to identify treatable traits for future studies. However, a weak effect was observed only in cases of marked hyperinflation. The reduction of *P*_tcCO_2__ could also lead to unloading the respiratory muscles and reducing the respiratory drive in patients with chronic hypercapnic respiratory failure [26]. COPD patients frequently suffer from muscle dysfunction, and recent data suggests a correlation of this phenomenon with elevated CO_2_ levels [27]. A number of pathophysiological studies demonstrated that NHF clears anatomical dead space in a flow-dependent manner, thereby reducing the re-breathing of expired gas rich in CO_2_ [28, 29]. This represents a key mechanism through which NHF enhances gas exchange and potentially reduces *P*_tcCO_2__ [30]. So far, there are no explanations why NHF reduced RR during wakefulness, while during sleep an increase in RR occurs under NHF in contrast to LTOT. An asymmetrical cannula interface may further enhance CO_2_ elimination, even at low NHF settings that may improve adherence, by generating reverse flow in the upper airways and reduced *V*′_E_, along with the lower WOB, as shown in hypoxaemic patients [31, 32].

The sleep quality in severe COPD patients is generally reduced, which was confirmed in the current study also, and this did not change following the home phase [33]. After 4 weeks of treatment within the home environment, the proportion of light sleep (sleep stage 1) was slightly lower under LTOT, but deep sleep and REM sleep were not adversely affected by NHF. This could be explained by the NHF-induced noise that patients may hear during light sleep. There was a significant reduction in CAT and SGRQ scores in the NHF group relative to LTOT and the baseline assessment completed before discharge. The scores were not different between the baseline and after 4 weeks of LTOT.

An increase in QoL by NHF could be biased due to the crossover study design, but the similar improvement was previously reported also and was not associated with improvements in pulmonary function tests or physical capacity [12, 34]. The positive effects of NHF in COPD patients could be due to various factors like the reduction in the WOB, CO_2_ or facilitated clearance of excessive airway secretion caused by hydration of the airway surface with humidified gas, positive expiratory pressure and expiratory resistance during NHF [13, 35]. The nocturnal *P*_tcCO_2__ may serve as a biomarker of improved gas exchange during respiratory support with NHF of hypercapnic COPD patients, and the measurement of *P*_aCO_2__ during the day might not provide sufficient information.

All COPD exacerbations occurred during the first treatment period, and the number of events was higher during LTOT (six *versus* two), consistent with the higher readmission rate of COPD patients soon after discharge following acute exacerbation. A smaller number of SAEs during the NHF period may potentially indicate a reduction in the exacerbation rate, but this should be studied in a randomised controlled trial. In a recent trial in stable hypercapnic COPD patients, Nagata *et al*. [36] showed a reduction of moderate/severe exacerbations by NHF. So far, there are no reliable data from which pathophysiological explanations for the observed reduction in the exacerbation rate can be derived. One possible explanation could be that breathing with warm and humidified gas during NHF maintains thermodynamic balance in the airways, preventing cooling of the epithelium. This benefits the antiviral immune defense function which is mediated by the excretion of nasal epithelial extracellular vesicles [37]. Breathing cold air is believed to be the mechanism of the seasonal variation in infection in the upper respiratory tract and may also explain the seasonality in COPD exacerbations and a reduction of the exacerbations by the use of long-term NHF [38].

### Strengths and limitations

First of all, this study was conducted at a single centre in one country, which limits its generalisability. As expected, there was a high dropout rate among patients, likely reflecting the high severity of disease or poor health status in this population. This high dropout rate highlights a real-life challenge in conducting trials involving very sick COPD patients immediately after discharge, particularly when broad inclusion criteria are applied. However, it also limits the statistical power, especially regarding secondary outcome measures such as QoL and exacerbation rates, from which inferences must be drawn with great caution. At the same time, an inclusion of patients who may potentially have early re-exacerbation is a key strength of the current study. This study was designed to provide guidance for future randomised controlled trials where NHF would be used mostly overnight, with a focus on reducing readmissions.

*V*_T_ during sleep studies was not measured, preventing an assessment of *V*′_E_ at night-time. *V*′_E_ was significantly reduced by NHF during wakefulness. It is known that *V*′_E_ is also reduced by NHF during NREM sleep by a decrease of *V*_T_, but whether it becomes lower in severe hypercapnic COPD patients when *P*_tcCO_2__ is reduced by NHF remains an open research question [23, 39].

The high-flow device logged the total recording time only, and the actual time when the patient used NHF therapy can be overestimated. The QoL results, as well as NHF rate preference, could be influenced by a placebo effect of the open-label crossover design. However, randomised controlled clinical trials in stable COPD patients also showed improvements in QoL scores over the longer period of NHF therapy [12, 34, 36]. The effect of NHF on exacerbation rate and QoL should be investigated in large patient populations.

The patients were included during the hospital stay immediately after severe exacerbation. An expected improvement of CO_2_ during the weeks after the exacerbation might dilute results, but in our population the CO_2_ levels remained constant over the home study period. Data from present literature indicate screening of patients with COPD after receiving acute NIV to identify persistent hypercapnia, which is defined as *P*_aCO_2__ >53 mmHg [40]. However, these recommendations were not yet recognised at the time of the study design. On average, the patients were hospitalised for 10 days, and patients whose *P*_aCO_2__ improved below 50 mmHg were excluded from the study (figure 1). We have designed the trial with the aim to reduce CO_2_ comparable to the attainable effects of a low intensive NIV therapy as has also been shown in a recent meta-analysis [41].

The results of the current study might not be extrapolated to all COPD patients: the study included hospitalised hypercapnic patients immediately after severe exacerbation. This patient group has a high-risk profile, low QoL, poor prognosis, a high readmission rate and very high health-related costs.

### Conclusion

NHF reduced *P*_tcCO_2__ during sleep without adversely affecting sleep quality in a group of mild hypercapnic patients with severe COPD. During wakefulness, NHF lowered the *V*′_E_ without affecting *P*_tcCO_2__. Randomised controlled trials are needed to confirm the observed reduction of exacerbation rate and the improvement in quality of life with NHF.

## Data Availability

The datasets used and/or analysed during the current study are available from the corresponding author on reasonable request.
